# Simulation of gap junction formation reveals critical role of Cys disulfide redox state in connexin hemichannel docking

**DOI:** 10.1186/s12964-023-01439-z

**Published:** 2024-03-18

**Authors:** László Héja, Ágnes Simon, Julianna Kardos

**Affiliations:** Institute of Organic Chemistry, Research Centre for Natural Sciences, Magyar Tudósok Körútja 2, 1117 Budapest, Hungary

## Abstract

Video Abstract

**Supplementary Information:**

The online version contains supplementary material available at 10.1186/s12964-023-01439-z.

Despite the fundamental functions of connexin gap junctions (GJs), the molecular mechanisms governing the formation of intercellular GJ channels (GJCs) by docking of hemichannels (HCs) remain poorly understood. In order to approach GJC formation, we built the Cx31.3 HC structure-based homology model of Cx43 HC and positioned two membrane-embedded Cx43 HCs at varying distances to simulate HC-HC docking. We revealed that the exceptionally high number of conserved Cys disulfide bonds at the extracellular interface play a pivotal role in HC docking. Explicitly, the opening of extracellular Cys disulfide bonds in the Cx43 HC-HC model resulted in the disappearance of trans-GJ stabilization centers (trans-GJ SCs), going up against GJC formation. We have shown that the presence of an adjoining HC contributed to extracellular Cys disulfide formation and consequently to the emergence of trans-GJ H-bonds. Analysis of the channel size during molecular dynamics simulations of the experimentally determined Cx43 HC, Cx43 HC-HC and Cx43 GJC structures also showed that closed disulfide conditions can be linked to functionally open state of the channel, while open disulfide conditions lead to reduced channel diameter.

These findings suggest that several Cx HC channels in vertebrates may undertake intercellular HC docking similarly and may bring forward the connexin-specific targeting of HC docking.

Members of connexin protein (Cx) family forms hemichannel hexamers (HCs, connexons), embedded in cellular membranes. The formation of intercellular gap junction (GJ) channels (GJCs) entails docking of HCs to their HC counterparts on the neighbouring cell. These channels serve solute transfer between adjoining cells and also play a role in specific linkage [1–4]. Despite the expanding knowledge on the cellular regulation, structure, operation and functions of GJ channels [5–30], the molecular mechanism of HC-HC docking are not well understood [31]. In general, GJC structures (see SI Table S1) are characterized by 36 conserved Cys disulfides extracellularly, cross-bonding extracellular loops 1 (EL1) and 2 (EL2). Primary studies revealed crucial role of these disulfide bonds in shaping GJ via pairing Cys(1)-Cys(3), Cys(2)-Cys(2) and Cys(3)-Cys(1) in EL1 Cys(1)XXXXXXCys(2)XXXCys(3) and EL2 Cys(1)XXXXCys(2)XXXXXCys(3) sequences [22, 30, 32–37]. In addition, Retamal and co-workers devised the redox sensor function of the extracellular Cys matrix in connexins [23, 38, 39], thus making a case of Cys thiol-disulfide oxidation and Cys disulfide exchange, considered subsequently.

GJCs have received attention due to their probable roles in various physiological and patho-physiological processes [40–53], such as epilepsy [54], Alzheimer disease [55] or cancer [56–58]. The lack of subtype-specific inhibitors for GJCs, however, has hindered the targeting of GJCs in pharmacological strategies. We previously demonstrated that even mimetic peptides, considered to be Cx-specific bind to various parts of EL1/EL2 sequences, instead of the specific region where they were designed to [59]. Hence, we considered switching over to an alternative approach to possibly achieve GJC subtype specificity by identifying non-conserved residues in patterns of protein stabilization centers (SCs) that build up the HC-HC interface via hubs of interactions.

In this study we aimed at molecular understanding of Cx HC docking by distinguishing *i*) the formation of trans-GJ SC patterns and trans-GJ H-bonds, *ii*) spontaneous Cys thiol → disulfide oxidation and Cys disulfide exchange in HC docking and, *iii*) channel size determining the functional open (ON) or closed (OFF) states of HCs and GJCs in relation to the redox state of extracellular Cys residues. We used the homomeric Cx43 GJC as prototype since it is abundant on astrocytes and play a crucial role in the development and regulation of neural circuit function and animal behaviour [54, 55, 60–65]. We created a Cx43 HC homology model based on Cx31.3 HC (PDB code: 6L3T; SI Table S1, SI Fig. S1A) [66]. To simulate HC docking, two membrane bilayer-embedded Cx43 HCs were paired according to the architecture of the homomeric dodecamer Cx26 GJC (SI Fig. S1) and the progress of HC docking was simulated by placing two membrane bilayer-embedded HCs at varying distances (Cx43 HC-HC). In addition, we analysed the channel size of the HC, HC-HC and GJC structures based on the recently published experimental Cx43 HC and GJC structures [67].

## Results

### Simulation of Cx43 HC docking: the development of trans-GJ SCs

**Fig. 1:**
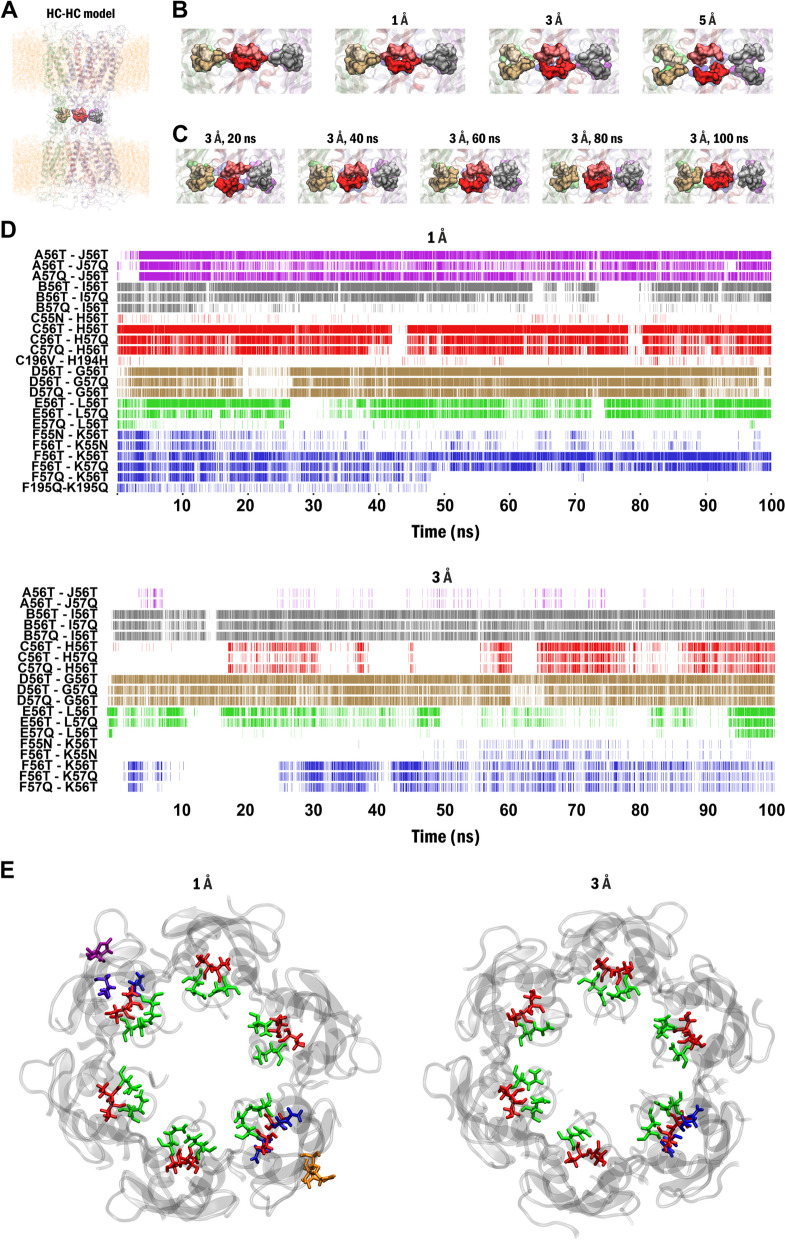
Spatio-temporal coordination dynamics of Cx43 HC-HC reveal the build-up of trans-GJ SCs as an indicator for HC docking. **A** Large-scale side view of two membrane embedded Cx43 HCs, homology modelled using the calcium-free Cx31.3HC template (PDB code: 6L3T; SI Table S1) [66] (Cx43 HC-HC). Residues 55N-56T-57Q of pre- and post-GJ subunits A-J, B-I, C-H, D-G, E-L and F-K are highlighted light- and dark purple, grey, red, brown, green and blue, respectively. **B** Close view of the interface residues 55N-56T-57Q of the Cx43 HC-HC model show gradual disintegration of the trans-GJ interface with increasing distance between HCs. **C** After moving HCs away to 3 Å distance, appearance of specific trans-GJ SCs during 100 ns MD simulation show rebuilding of trans-GJ SC interactions. SCs defined at the A-J, B-I, C-H, D-G, E-L and F-K interfaces are highlighted in purple, grey, red, brown, green and blue, respectively. Trans-GJ SC pairs are designated as subunit name + residue number + 1-letter residue name. **D** Dynamics of trans-GJ SCs during the 100 ns MD simulation after moving HCs away to 3 Å distance demonstrate rebuilding of trans-GJ interactions. Note the development of trans-GJ SCs between subunit pairs C-H (light and dark red in C). **E** Residues forming trans-GJ SCs in top view of the Cx43 HC-HC models in the last 10 ns of MD simulations. Blue: 55N, red: 56T, green: 57Q, purple: 194H, orange: 195Q, violet: 196V

To understand GJC formation from HCs and to identify regions and structural motifs playing pivotal roles in the process, we first homology-modelled Cx43 HC, based on the high-resolution cryo-EM structure of calcium-free Cx31.3 HC [66] (SI Table S1), the only available experimental Cx HC of the Cx family at that time. To simulate Cx43 HC docking, two Cx43 HC homology models, embedded in explicite membrane, were positioned face-to-face according to the arrangement observed in Cx26 GJC (Cx43 HC-HC) (Fig. 1A). This model was compared to the recently published experimental Cx43 GJC structure [68]. We showed that no significant differences can be observed (SI Fig. S2).

After setting up the initial paired Cx43 HC (Cx43 HC-HC; Fig. 1A), we moved the two HCs away from zero distance to 1 Å, 3 Å and 5 Å to model different stages of the HC docking process (Fig. 1B). All models were subjected to 100 ns MD simulations and we continuously monitored the appearance of SCs from the two adjoining HCs (trans-GJ SCs), previously shown to build up the Cx43 HC-HC interface [69]. When HCs were positioned at their original locations or at a distance of 1 Å (*see* Methods section), the HC-HC interface formed by residues 55N-56T-57Q [69] were found to be stable (Fig. 1B). Increasing the distance to 3 Å and 5 Å introduced a significant and increasing gap between the two HCs even at the beginning of the simulation (Fig. 1B). During the 100 ns simulation, trans-GJ SC patterns of the Cx43 HC-HC interface reappeared when the original HC-HC distance was set to 3 Å (Fig. 1C).

To quantitatively address the progress of Cx43 HC-HC coupling, we investigated the appearance and progressive emergence of trans-GJ SC patterns, considered as indicators for successful HC docking. Several trans-GJ SC patterns could be identified between the internal 55N-56T-57Q-58Q and external 194H-195Q-196V EL1 and EL2 residues, developing interface between the two opposing HCs (Fig. 1A). When the two HCs were positioned at 1 Å distance, these interactions remained intact (Fig. 1D). However, once the distance was increased to 3 Å, many of these trans-GJ SCs disappeared, suggesting partial docking. Importantly, several trans-GJ SCs were found re-establishing during the 100 ns simulation (Fig. 1D, E). These findings demonstrate that the MD of pre-positioned single HCs can be an appropriate model of HC-HC coupling and GJC formation.

We were able to capture the appearance of trans-GJ SCs at both inner (channel-facing) and outer (gap-facing) GJ surfaces. The abundant EL1 SCs at the inner surface consisted of extracellular loop EL1 residues 55N-56T-57Q, while the less numerous SCs at the outer surface were made of EL2 residues 194H-195Q-196V in each paired Cx43 HC-HC subunits. Interestingly, increasing the HC-HC distance to 5 Å resulted in irreversible and almost complete loss of trans-GJ SCs, leaving only a few trans-GJ SCs comprising bulky residues, 194H and 196V residues to persist (SI Fig. S3).

By investigating what structural motifs may contribute to the proper orientation of these residues, we noticed that 55N-56T-57Q and 196V were associated with the 54C(1)-198C(3) Cys disulfide bridge, while the sequential trans-GJ SC residues 194H-195Q conjoin the 61C(2)-192C(2) Cys disulfide bridge (SI Fig. S4). Some of these SCs correspond to H-bonding (51A “O”-201S “OG”; 53R “O”–199F “N”; 55N “N”-197D “O”; 58Q “NE2”-193P “O”) while others represent Van der Waals contacts between carbon atoms (SI Table S2). Three-dimensional structure of the HC-HC interface explains quasi-mirror arrays of tetragonal SC patterns (SI Fig. S4B), apparently oriented by Cys disulfide-linked trans-GJ SC and H-bond interactions (red lines) (SI Fig. S4C). Beside the trans-GJ SC contacts, the pattern of Cys disulfide linked intra-HC SCs is shown by SI Fig. S4D. The unique 65C(3) centred three dimensional SC pattern (SI Fig. S4) exhibits GJ contacts within and between EL1 and EL2 loops.

### Opening of Cys disulfide bonds abolishes trans-GJ SCs

**Fig. 2:**
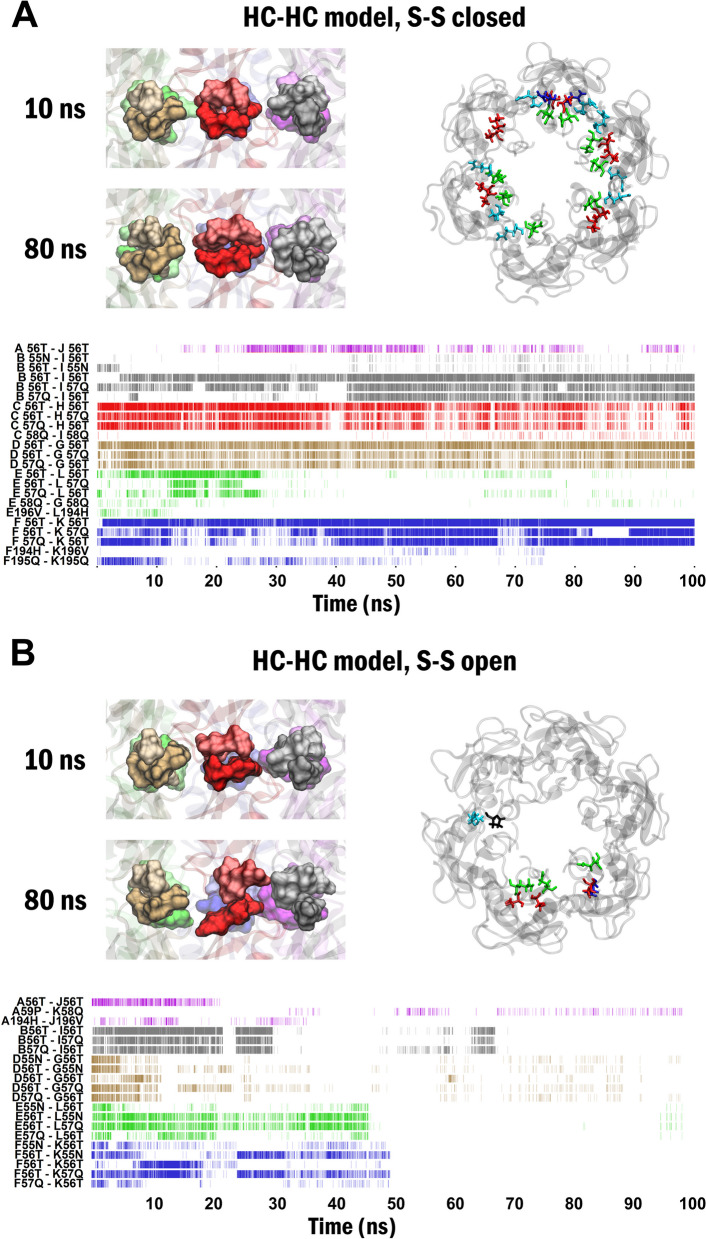
Distinguishable spatio-temporal coordination dynamics of Cx43 HC-HC model under closed *versus* open Cys disulfide bond preconditions. **A** Close view of the interface residues 55N-56T-57Q-58Q during 100 ns MD simulation (top left), top view of residues forming trans-GJ SCs in the last 10 ns MD simulation (top right) and dynamics of trans-GJ SCs (bottom) in the Cx43 HC-HC model in the closed Cys disulfide configurations. **B** Close view of the interface residues 55N-56T-57Q-58Q during 100 ns MD simulation (top left), top view of residues forming trans-GJ SCs in the last 10 ns MD simulation (top right) and disappearance of trans-GJ SCs (bottom) in the Cx43 HC-HC model in the open Cys disulfide configurations. Residues of trans-GJ SCs 55N-56T-57Q in pre- and post-GJ subunits in A-J, B-I, C-H, D-G, E-L and F-K pairs are highlighted purple, grey, red, brown, green and blue, respectively. Trans-GJ SC pairs are designated as subunit name + residue number + 1-letter residue code. Residue colors: 55N, red: 56T, green: 57Q, cyan: 58Q, black: 59P

Given the abundance of Cys disulfide bonds in the neighbourhood of the trans-GJ SCs and the ability of protein Cys thiols to spontaneously transform to Cys disulfides [70–74], particularly in connexins [23, 73, 75] we investigated whether Cys disulfide bond opening and closing may contribute to the transition from HC to GJC structures. To assess this hypothesis, we opened Cys disulfide bonds and applied 100 ns MD dynamics on the Cx43 HC-HC model at zero or intermediate (3 Å) distance between the two HCs. The assessment of spatio-temporal dynamics with zero HC-HC distance demonstrated that the opening of Cys disulfide bonds resulted in the disappearance of all trans-GJ SCs during the 100 ns MD simulation (Fig. 2). At the intermediate distance (3 Å), no trans-GJ SCs were developed during the 100 ns MD, in contrast to the closed Cys disulfide condition (Fig. 1C). Notably, inter-subunit SCs, stabilizing the Cx43 HC design remained unchanged (SI Fig. S5), demonstrating that opening of Cys disulfide bonds specifically affected the trans-GJ SC patterns. In summary, these results show that opening of Cys disulfides in the HC structure prevents trans-GJ SC pattern formation and displays characteristic HC design, conjuring Cys disulfide exchange by HC docking.

### Are disulfide bonds opened in the HC structure?

The presence of Cys disulfide bonds can only be assumed from the experimental structures. Although all currently available GJC structures contain extracellular Cys disulfide bonds, this could be a result of experimental conditions used in structure determination (SI Table S1). We have shown that trans-GJ SC patterns disappear after opening the Cys disulfide bonds, in accordance with experimental data [32, 39]. However, solo HCs can be fully functional even when all extracellular Cys disulfides were reduced [76], suggesting that Cys disulfides are crucial only in the GJC, but not in the HC form [39]. To explore whether Cys disulfides are closed or open in the solo HC, we compared the Cx43HC model based on the Cx31.3 template that represent the solo HC with the A-F subunits of the Cx43GJ model that represents the structure of the HC as it exists in a full GJC. Finally, we also compared the open Cx43 HC model to the Cx43 HC-HC model to assess the effect of constraints induced by the close presence of the opposing HC. All structures were subjected to 100 ns all-atom MD simulations with Cys disulfide bonds kept closed or opened at the beginning of the MD run.

**Fig. 3:**
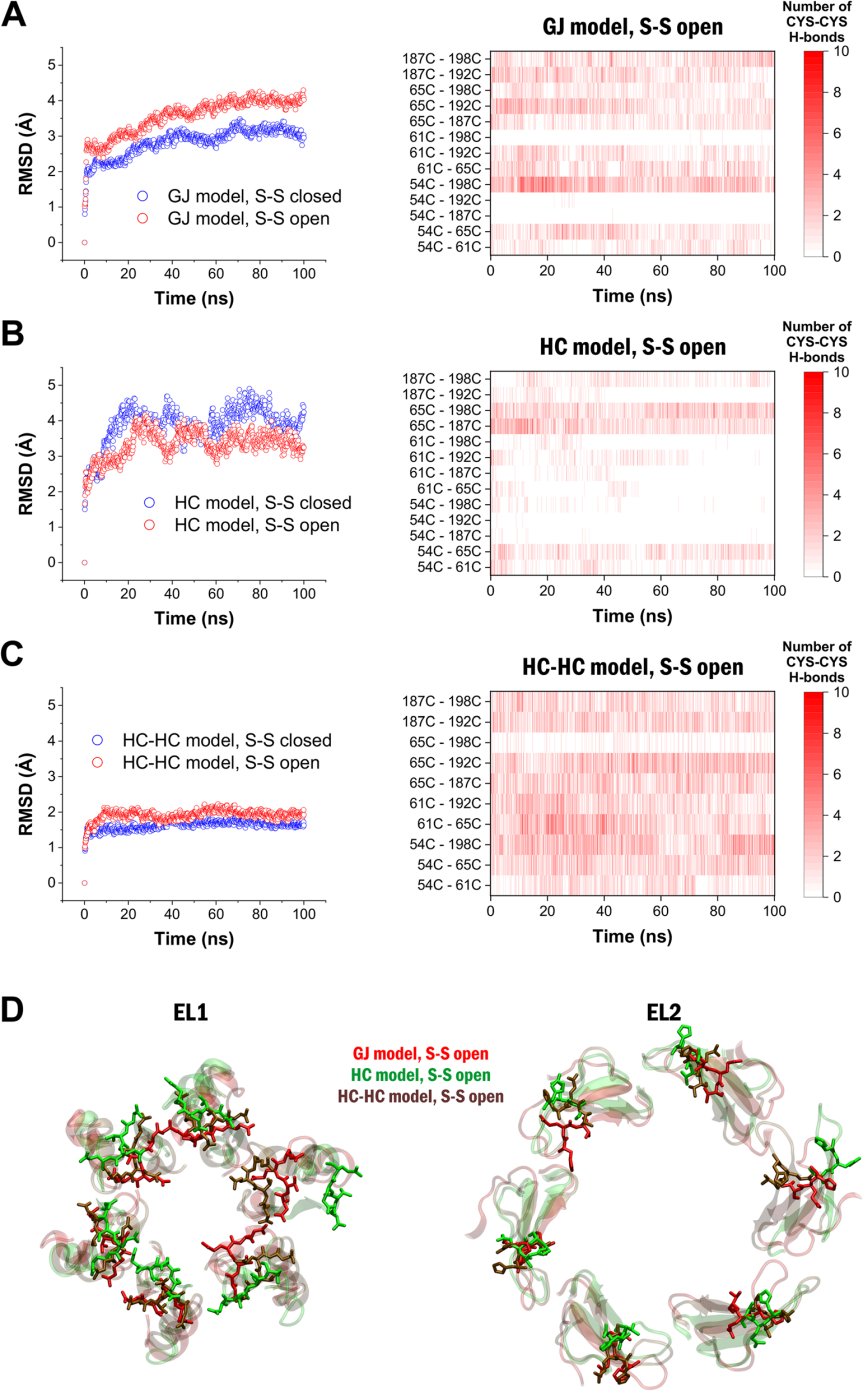
The structure of open solo HC model may be better represented by open Cys disulfide bonds. **A** RMSD changes of the extracellular region compared to the initial structure (left) and number of specific H-bonds between Cys residues during the 100 ns MD (right) in the Cx43 GJC model. **B** RMSD changes of the extracellular region compared to the initial structure (left) and number of specific H-bonds between Cys residues during the 100 ns MD (right) in the Cx43 HC model. **C** RMSD changes of the extracellular region compared to the initial structure (left) and number of specific H-bonds between Cys residues during the 100 ns MD (right) in the Cx43 HC-HC model. In order to compare HC model with GJC and HC-HC models, only the number of H-bonds on the A-F subunits were counted. Theoretically, two H-bonds can be formed on each subunit since Cys residues can be either proton donors or acceptors, totaling a dozen of H-bonds between Cys residues on all subunits. **D** Extracellular view of the EL1 (left) and EL2 (right) regions of the A-F subunits of the Cx43 GJC (red), Cx43 HC (green) and Cx43 HC-HC (brown) models with open Cys disulfide bond at the end of the100 ns MD runs. Extracellular loops are shown in cartoon representation, interface residues (55–58 and 194–196) are shown in stick

We found that in the Cx43 GJC model, significantly less conformational changes are required to reach a steady-state structure in the closed Cys disulfide configuration compared to the open Cys disulfide configuration, suggesting that closed Cys disulfide bonds are, indeed, representing the typical state of Cx43 GJC (Fig. 3A). In contrast, in the solo Cx43 HC model, despite of the higher degree of freedom, the open disulfide configuration approaches the steady-state structure more rapidly, implying that open disulfides may represent the physiological conformation of the solo HC (Fig. 3B). However, presence of the opposing HC in the Cx43 HC-HC model results again in significantly less conformational change in closed Cys disulfide configuration compared to the open Cys disulfide one (Fig. 3C). Comparisons of top views of open Cys disulfide Cx43 HC-HC, solo Cx43 HC and Cx43 GJC models indicate that the open Cys disulfide Cx43 HC-HC is markedly different from the solo Cx43 HC, instead, it is similar to Cx43 GJC (Fig. 3D). These data suggest that reduced Cys disulfides may be the more appropriate condition under which solo HCs exist, but the presence of the opposing HC may shift the structure to the Cx43 GJC architecture, a state in which oxidized Cys disulfides are more favored.

In addition, we also analyzed what Cys-Cys H-bond interactions show up in the open Cys disulfide configurations during the simulation, to estimate the possibility of the formation of different Cys disulfide bonds. According to this analysis, in the Cx43 GJ model, many different potential disulfide bonds can be formed throughout the MD run, including the 54C(1)-198C(3), 61C(2)-192C(2) and 65C(3)-187C(1) Cys disulfide bonds, present in the experimental Cx26 structure (Fig. 3A). These data show that despite opening the disulfide bonds at the beginning of the MD run, they can be easily rearranged. In contrast, opening the Cys disulfide bonds in the Cx43 HC model led to structural changes that opposed Cys disulfide formation by keeping Cys residues far from each other to bind (Fig. 3B). In the Cx43 HC model, the original 54C(1)-198C(3), 61C(2)-192C(2) and 65C(3)-187C(3) disulfide bonds are almost completely missing. The Cx43 HC-HC model was found to be similar to the GJC model, enabling all the original and many other Cys disulfide bond formations (Fig. 3C), confirming that the presence of opposing HC leads to restructuring of solo HC and favors Cys disulfide formation.

Conclusively, comparison of Cx43 GJC, solo HC and HC-HC models suggest that open Cys disulfide bonds may be associated with the HC form, but Cys residues are oxidized in the full GJC.

### Presence of reduced Cys disulfides in HC prevents H-bond interactions at the HC-HC interface

Our data showed that solo HC structure is consistent with open disulfide bonds (Fig. 3). However, opening of extracellular Cys disulfide bonds in the Cx43 HC-HC model resulted in the disappearance of trans-GJ SCs (Fig. 2B). These results are in line with previous observations showing that closed disulfide bonds are necessary for GJC formation, but are not required for the solo HC functioning [39, 76].

**Fig. 4:**
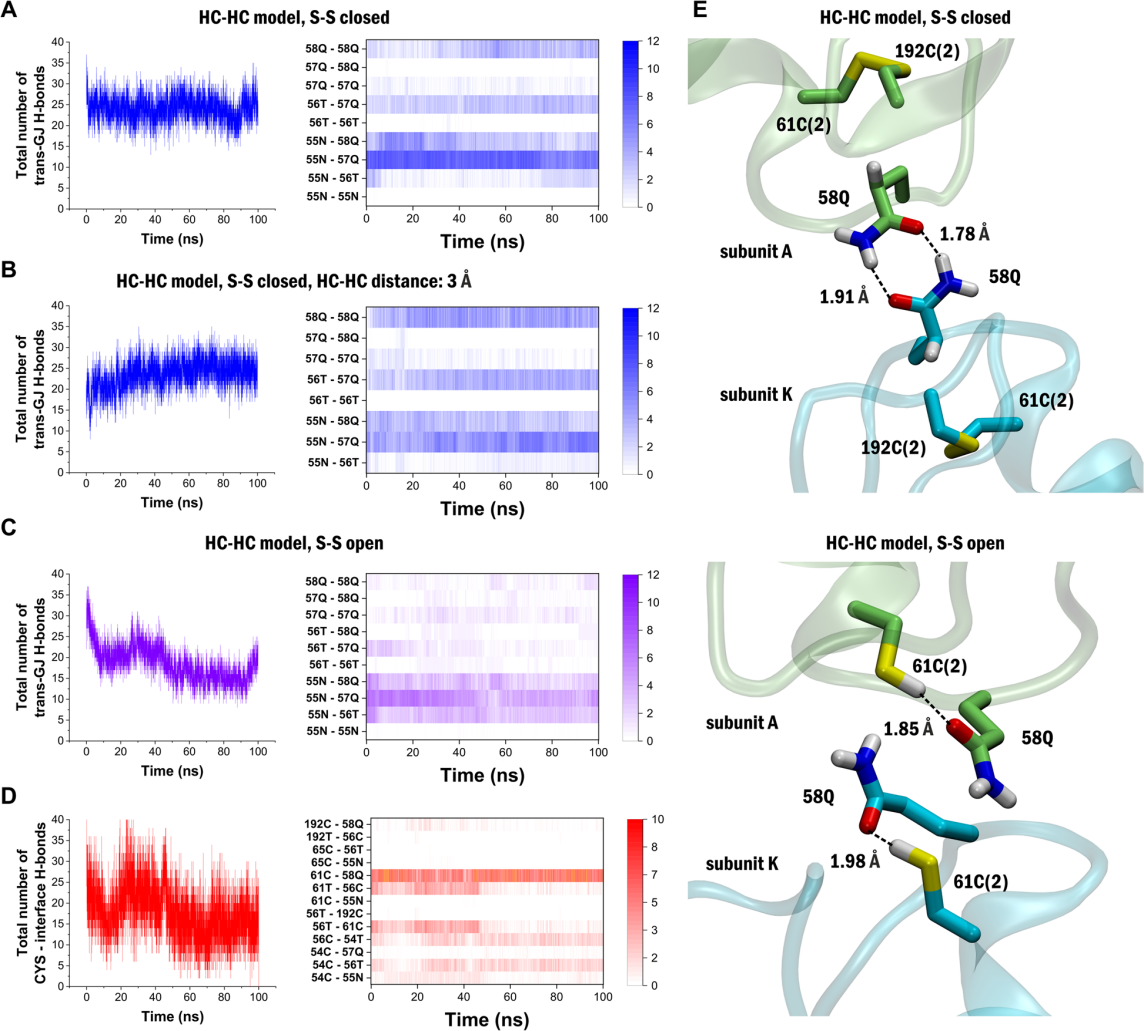
Free cysteine residues 54C, 61C and 192C in the S–S open model disorient residues involved in trans-GJ interactions. **A** Number of trans-GJ H-bonds (left) and specific residue pairs forming these trans-GJ H-bonds (right) during the 100 ns MD in the HC-HC model with closed disulfides. Colour bar shows the number of subunit pairs on which the specific trans-GJ H-bonds is present. **B** Number of trans-GJ H-bonds (left) and specific residue pairs forming these trans-GJ H-bonds (right) during the 100 ns MD in the HC-HC model with HCs positioned 3 Å away. Colour bar shows the number of subunit pairs on which the specific trans-GJ H-bonds﻿ is present. **C** Number of trans-GJ H-bonds (left) and specific residue pairs forming these trans-GJ H-bonds (right) during the 100 ns MD in the HC-HC model with open disulfides. Colour bar shows the number of subunit pairs on which the specific trans-GJ H-bonds﻿ is present. **D** Number of H-bonds involving Cys residues (left) and specific residue pairs forming these H-bonds (right) during the 100 ns MD in the HC-HC model with open Cys disulfides. Colour bar shows the number of subunit pairs on which the specific Cys-interface H-bond is present. **E** 3D representation of the HC-HC interface shows disorientation of 58Q from the position required to make trans-GJ H-bonding. Trans-GJ H-bonds between opposing 58Q residues are formed in the HC-HC model with closed disulfides (top). In the open disulfide model (bottom) 58Q oxygen atoms are involved in H-bonds with Cys thiol residues instead of forming trans-GJ interactions

To assess what structural changes may underlie the transition process, we explored which residues are involved in the interactions between the two HCs. We observed that trans-GJ H-bonds are formed between residues 55N, 56T, 57Q and 58Q in the closed Cys disulfide HC-HC model (Fig. 4A). The same interactions emerged during the MD simulation when the two HCs were initially positioned at 3 Å distance (Fig. 4B). Some of these interactions, especially those forming between 58Q residues on both HCs disappeared when the Cys disulfide bonds were open (Fig. 4C). Analyzing the structural changes corresponding to the in silico reduction of Cys disulfide bonds, we found that the Cys thiol residues in the open Cys disulfide model can form intra-subunit H-bonds with various residues involved in trans-GJ interactions, thereby weakening the potential of HC-HC docking (Fig. 4D). For example, intra-subunit H-bonding between 61C(2) and the oxygen atom of 58Q prevents the formation of H-bonds between the opposing 58Q residues (Fig. 4E). Therefore, keeping the HCs in open Cys disulfide configuration inhibits HC docking and GJC formation.

### Potential functional consequences of disulfide opening

Since HC, but not GJC structure have been shown to be consistent with open disulfide conditions, we intended to investigate whether the reduced or oxidized states of Cys residues may be associated with functional open or closed states of the connexin channels. As discussed above, extracellular Cys residues have been suggested to act as redox sensors [39] and it has been shown that HC and GJC channel functions are differently affected by the redox environment [76].

To explore the functional state of connexins in the open and closed disulfide conditions, we analysed the channel diameter of the recently published experimental Cx43 HC structure (Cx43 HC) [67], a full channel constructed from two opposing Cx43 HCs (Cx43 HC-HC, *see* Methods) and the experimental Cx43 GJC [67]. All of these structures were subjected to MD simulations during which the size of the water-accessible channel was determined using the HOLE suite of tools [77, 78]. Although open (ON) and closed (OFF) HC and GJC channel functions are generally suggested to be governed by an intracellular “chain-and-ball” gating mechanism [79], we intended to measure whether size of Cx channels may be affected by oxidization state of conserved extracellular cysteines.

**Fig. 5:**
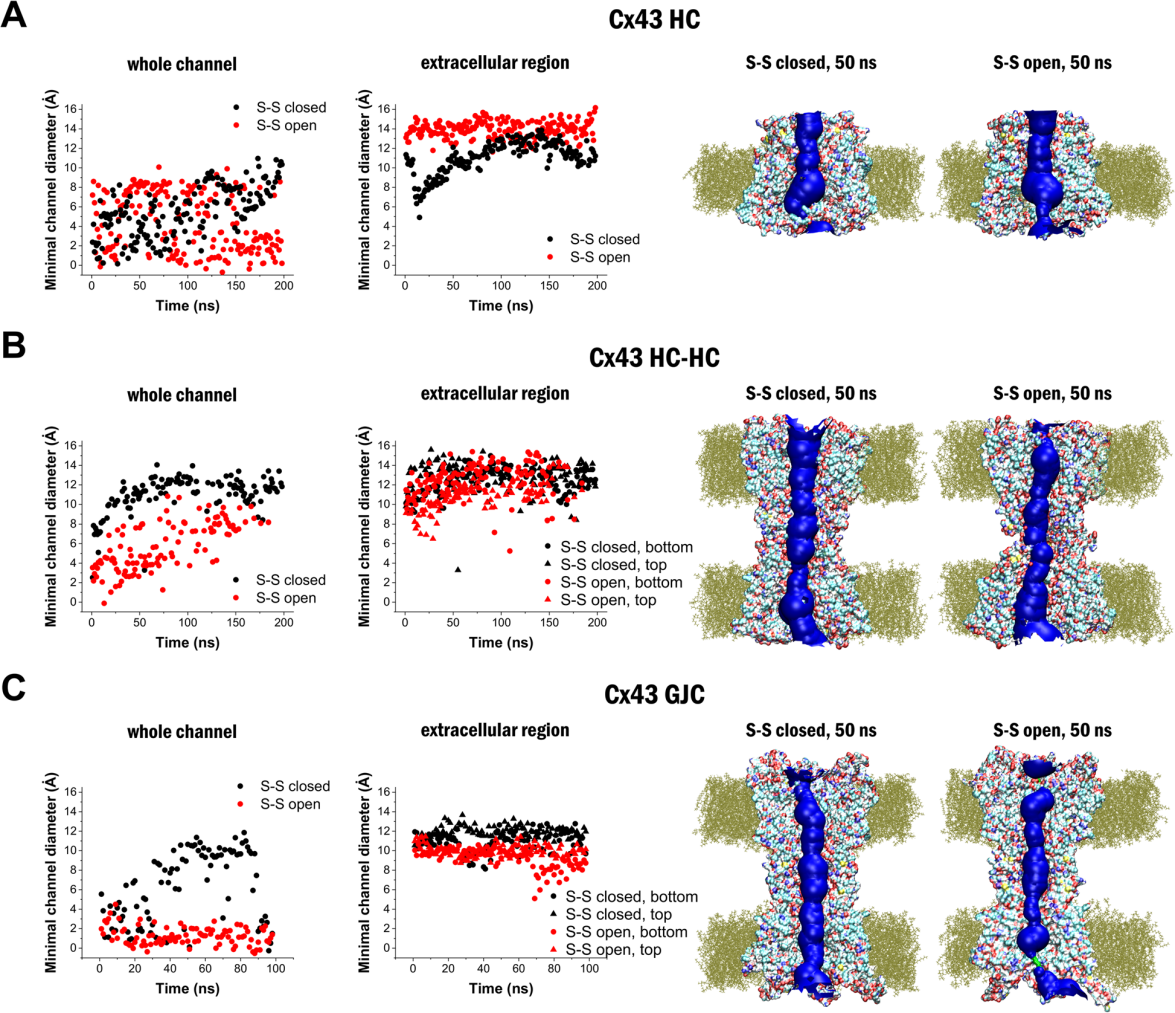
Closed and open disulfides can be related to open (ON) and closed (OFF) channel functions. Dynamics of channel diameter in the whole channel and specifically in the extracellular region (left) and visualization of the water accessible channel as determined by the HOLE software after 50 ns MD simulation (right) in the Cx43 HC structure [67] (**A**), in the HC-HC model constructed from the Cx43 HC structure [67] (**B**) and in the Cx43 GJC structure [67] (**C**). Water accessible channel is visualized as blue shape, lipids are shown in olive, Cx residues are colored according to atom type. Lipids and connexins are sectioned for better representation of the water accessible channel

We observed that in the Cx43 HC structure (Fig. 5A), channel size was characterized by alternation of the minimal channel diameter between approximately 2 Å, representing the fully closed OFF state and approximately 8 Å, observed in the semi-permeable ON state [67, 68], that allows the transfer of Na^+^ and K^+^ ions with their first hydrate shells [80]. Importantly, size of the HC channel was not significantly affected by the redox state of the Cys residues (Fig. 5A), in accordance with the experimental observation showing that HC channels are equally functional in both reducing and oxidizing environments [81]. In contrast, building of a HC-HC channel pair from the same experimental Cx43 HC channels greatly differentiated between open and closed S–S forms (Fig. 5B). In the HC-HC structure, channel size increased from 7 Å to 12 Å during the 200 ns MD simulation when the S–S bonds were closed. These values are associated with the functionally open channel [68, 81]. By contrast, channel size in the open S–S bond configuration changed in the range of 3–9 Å, associating closed or partially closed OFF channel function (Fig. 5B). Importantly, the results obtained for the HC-HC construct was also confirmed and validated by MD simulations of the experimental Cx43 GJC structure [67], which clearly showed that the primary open state of the GJC channel is attributed to the closed S–S state and is incompatible with the open S–S configuration (Fig. 5C). These results further support that closed S–S configuration is corresponding to the functionally open ON channel, while open S–S configuration is in accordance with the functionally closed OFF channel, which is the resting state of the solo HC form. It is also worth mentioning that although the Cys residues are located in the extracellular region, reduction of the channel size was most prominent near or in the intracellular region (Fig. 5 right).

## Discussion

In this study, we simulated intercellular connexon coupling by pairing two membrane-embedded Cx43 HCs. The simulations revealed consecutive building-up of trans-GJ SCs. By investigating the structural prerequisites of HC docking we revealed that residues of the internal EL1 55N-56T-57Q-58Q and external EL2 194H-195Q-196V HC-HC interface in Cx43 are oriented to the appropriate conformation by the large number of neighbouring Cys residues. By exploring the structures of Cx43 HC and Cx43 GJC or Cx43 HC-HC models, we found that the HC form may also be coherent with open Cys disulfide bonds. We also revealed that open disulfide bonds may be attributed to the functionally closed (OFF) state of connexin channels. These observations provide mechanistic clues that relate gap structure and HC docking through the extracellular Cys residue-linked development of subtype-specific trans-GJ interactions.

The emergence of Cys in SC patterns with Arg/Lys in the gap as characterized by minimum distances 3.34 Å and 3.23 Å in 65C(3)-189Arg and 187C(1)-68Lys contacts, respectively (SI Table S2), raises the issue of HC docking via oxidative Cys disulfide exchange [32, 70, 71, 73–75] activated by positively charged flanking Arg or Lys [82–86]. As being part of the cause of HC docking, we may conjecture Cx redox sensor functioning [23, 38, 39, 75, 87, 88] and GJC formation. Noteworthily, Cx43 expressed in plasma membrane of *Xenopus oocytes* without Cys residues did not form GJC but HCs [76].

Now the matter arises whether disulfide reversibility [72] may be assigned to Cx HC docking. In line with previous suggestions, the low pKa Cys thiol may spontaneously oxidize to Cys disulfide at neutral *p*H generating two protons in addition to two electrons [83]. The process involves the formation of reactive thiolate anions that oxidize to sulfonic acid thus elevating reactivity towards nearby Cys thiol groups [89]. Having a correlation time in the 10^–7^ to 10^–9^ s range [90], the rapid proton transfer to flanking Arg/Lys can further Cys disulfide bond formation. We put forward the proton-acceptor feature of Arg/Lys that enhances the redox-sensitivity of Cys residues along with the rate of thiol/disulfide exchange. Running MD simulations in the 100 ns time range we noticed fast fluctuations of nearest 68Lys/189Arg residue distances from Cys in the range mainly similar with that of 65C(3)–187C(1). The time to distance variations mostly compare with 1–5 ns Cys disulfide bond exchange events simulated for 35 ns in the β3 integrin subunit [91].

**Fig. 6:**
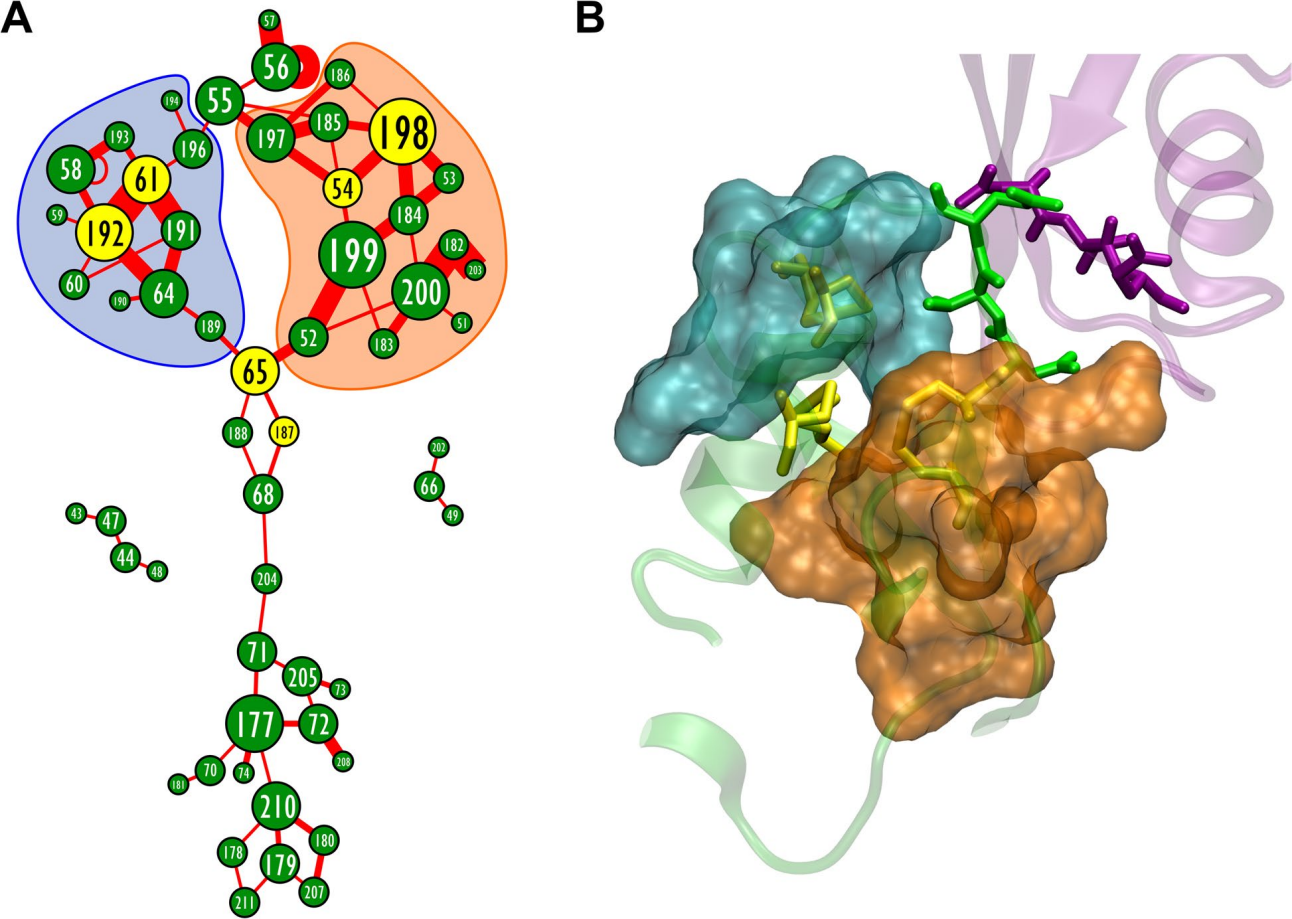
Presumed Cys disulfide exchange controlled formation of stabilization centers (SCs) orients Cx43 HC docking. **A** Graph representation of extracellular SCs in the GJC built from two opposing HCs after distance between the HCs set to 3 Å. Nodes represent residues, edges represent SCs formed between given residues. Data of all subunits during the whole 100 ns MD run is summarized into a single graph. Size of the nodes corresponds to the number of SCs the given residue is participated in. Width of the edges corresponds to the stability of the given SC during the 100 ns MD run. Cys residues are highlighted by yellow nodes. **B** 3D structure of subunits A (green) and J (purple) in the GJC built from two opposing HCs after distance between the HCs set to 3 Å. Interface residues (55N-56T-57Q) are shown in green and purple stick representations on the A and J subunits, respectively. Cys residues are shown in yellow stick representation. SC associations identified in A are shown in cyan and brown surf representations

To investigate the structural patterns and potential role of extracellular SCs in GJC formation, we visualized the connectivity of SCs in the HC-HC model, distanced at 3 Å using a graph representation of all data of all subunits during the entire 100 ns MD run (Fig. 6A). The connectivity pattern revealed that two Cys-centred patterns (moduls) can be distinguished that are connected by the central node 65C(3). The two patterns, stabilized by the 54C(1)-198C(3) and the 61C(2)-192C(2) disulfides, both consist of SCs involving the EL1 and EL2 loops, highlighting the importance of EL1-EL1 and EL2-EL2 interactions [92–94]. These modules, as well as the group of trans-membrane exits of TM2, TM3, TM4 helices are connected by the 65C(3)-associated pattern of SCs (Fig. 6A). Another SC pattern comprising the 43S-47D, 44A-47D, 44A-45W residues involved in Ca^2+^ binding by 43S, 46E and 48E. A further independent SC pattern is characterized by 66Y edging 49Q and 202R, which assists positioning of TM1 and TM4 in concert with molecular changes occurring in the TM2-connected extracellular helix. Apparently, the graph derived from SC dynamics describes that two Cys-centred patterns of SCs, connected by the conserved sequence 65C(3)-66Y-67D of the extracellular helix, form the “gap syntone” that guides proper lining of 55N-56T-57Q for HC-HC coupling (Fig. 6B). The central role for 65C(3) contacting 189R may allow the prevention of GJC design by binding guanine to 189R [95]. Our results also suggest that other SC patterns emerging from coupling at the TM helix-outer membrane interfaces indicate dynamic changes in the local lipid environment. Indeed, all-atom MD simulations of cryo-EM data from the native Cx46/50GJ in lipid discs reveal the lipid-induced stabilization of the GJC and vice versa [96].

Spatio-temporal configuration dynamics of Cx43 HC docking enable the observation of atomic scale development of extracellular Cys-linked trans-GJ SCs between EL1 C(1)vvzvvv(2)zzzC(3) and EL2 C(1)zzzvC(2)vzzvvC(3) sequences, in which 100% of C(1), C(2), C(3), > 80% of X residues (v) and < 80% of X residues (z) are conserved [69]. In view of conserved extracellular Cys residues and extensively homologous protein sequences of Cx isoforms, along with their mutations, phosphorylated and ubiquitinated derivatives [42, 45, 53, 97, 98], it seems likely that several homo- and heteromeric Cx GJCs in vertebrates are formed according to similar patterns. The less conserved extracellular residues do introduce subtype-specificity and may also confer non-docking nature, as observed in Cx31.3HC [99].

Our trans-GJ SC interface design may serve the development of subtype-specific inhibition of intercellular HC docking by targeting the build-up of trans-GJ SCs. It seems feasible by triggering trans-GJ SC site-specific targeting of inhibitory ‘substrate’ by adjoining cysteine-targeted electron withdrawing group [100–102] through an actual linker. Meaningfully, the substrate-adjunct design may expect Cx43 subtype-specific inhibition by applying 55H-56T-57Q isosteric non-peptide substrate derivatives. Our perspective also anticipates that the alleged substrate-adjunct design may overcome potential off-target interactions [103, 104] by enhancing the intrinsic reversibility of the electron-withdrawing group [100]. Also, the fine tuning of span and flexibility of the linker can serve selectivity of substrate-adjunct inhibitors. Even now, the subtype-specific inhibition of HC docking could elicit side-effects in the molecular mechanisms of actual pathologies. For example, decoupling of astrocytes via inhibition of Cx43 HC coupling may induce the impairment of synaptic plasticity and spatial learning memory [105]. We place this issue in the context of cell-specific inhibitor delivery [106].

## Methods

### Generation and molecular dynamics of Cx43 HC model

The Cx43 HC was prepared using the high-resolution cryo-EM calcium-free Cx31.3 HC structure as a template (PDB code: 6L3T) [66]. Swiss-Model generated an alignment between the target and the template (SI Fig. S1A) and built the calcium-free Cx31.3 HC (6L3T) template-based model. After a short energy minimization, we obtained the Cx43 HC model. This was submitted to the „Positioning Proteins in Membrane” (PPM) server of the „Orientations of the Membranes in Proteins” (OPM) database [107] to predict the trans-membrane regions 20–46, 74–94, 154–176, 205–226, and to rotate the protein parallel to the z axis. The Cx43 HC model was then subjected to 100 ns molecular dynamics (MD). In detail: The model was loaded to the workspace of Maestro-Desmond (D. E. Shaw Research) [108]. The protein preparation wizard was invoked and pre-processing was performed with the “create disulfide” option checked or unchecked depending on the simulation conditions. This option ensures that Cys residues remain in disulfide-bond state all over the simulation or Cys residues are present as thiols. Also, H-bonds were added during pre-processing. A predefined membrane was added and “placed on the pre-aligned structure”. Since the predefined membrane is positioned in the *x–y* plane, the OPM-rotated protein was aligned accordingly, perpendicular to the membrane. The structure was subsequently loaded into the Desmond module. Temperature and pressure were kept constant at 300 K and at 1 atm pressure, respectively (NPT condition). Simulation time was set to 100 ns, the recording interval was set to 10 ps, so altogether 10 000 frames were collected.

### Generation and molecular dynamics of Cx43 HC-HC model

Two copies of the solo Cx43 HC model (10,824 atoms each) were taken. One copy was aligned to subunits A-F of the template Cx26 GJC with closed Cys disulfide bonds (PDB code: 2ZW3; SI Table S1) [109] with Pymol. The other copy was aligned to subunit G-L of the same template. Prior to aligning, the second copy was renumbered, to start from atom No. 10825, to provide continuous atom numbering in the newly generated dimer. This resulted in a raw model of the docked Cx43 HC, in which several interface residues were positioned in a clashing distance of less than 2 Å. Therefore, a short minimization was performed in Maestro on all atoms, using the “minimize all” command. Subsequently, the model was submitted to OPM, and the Cx43 HC-HC model was prepared in visual MD (VMD), because VMD allows the application of two membrane bi-layers per protein [110]. First, to generate protein structure files (psf) the structure was split into 12 individual subunits. Cys disulfides were set between 54C(1) and 198C(3), 61C(2) and 192C(2) along with 65C(3) and 187C(1) using the DISU (disulfide) patch of VMD. Also, an open version was prepared, without creating Cys disulfide bonds. The 2 × 6 apposed subunits were then combined into a single file and two POPC membrane bilayers (150 × 150 Å each) were generated by the membrane builder plugin. Both HCs were embedded in membrane and the whole system was solvated as described previously [69]. The “keep water out” tcl script was modified in-house to be appropriate for two membranes [69]. Simulation time was set to 100 ns, the recording interval was set to 10 ps, so altogether 10 000 frames were collected.

### Generation and molecular dynamics of Cx43 HC-HC model with varying HC-HC distance

The paired Cx43 HC-HC model was taken as a starting structure for HC docking. The protocol was the same as above until the first POPC lipid was put on one HC (subunit G-L) and a combined lipid-protein pdb file was saved along with its protein structure file (psf). After this stage, subunits A-F were selected and moved by 1 Å in the z direction using the “moveby” command of VMD that moves each of the selected atoms by the given vector offset. After lifting all atoms of one of the Cx43 HCs by 1 Å, all atoms of the POPC lipid membrane around the Cx43 HC were also moved by 1 Å in the z direction and saved together with the HC containing subunits A-F. Then the protocol continued as described above. To follow the effect of HC-HC distance in a stepwise manner, three different distances were introduced: 1 Å, 3 Å and 5 Å**.** Disulfides were kept closed in all cases and the simulation lasted for 100 ns.

### Generation and molecular dynamics of Cx43 GJC model

The Cx43 GJC model was built based on the Cx26 GJC structure [109] as described before [69]. Briefly, an initial model was built by Swiss-Model, which was submitted to OPM to predict TM regions. Subsequently, the full Cx43 GJC model was placed in two POPC membrane bilayers as in the Cx43 HC-HC model. The Cx43 GJC model was prepared with closed and open Cys disulfide conditions as was with the Cx43 HC-HC model. MD was performed as above for 100 ns.

### Preparation and molecular dynamics of the cryo-EM based Cx43-HC structure

Cx43 HC based on the recently published cryo-EM structure [67] (PDB code: 7Z23) was prepared in visual MD (VMD),. First, to generate protein structure files (psf) the structure was split into 6 individual subunits. Cys disulfides were set between 54C(1) and 198C(3), 61C(2) and 192C(2) along with 65C(3) and 187C(1) using the DISU (disulfide) patch of VMD. The open S–S version was prepared without creating Cys disulfide bonds. The structure was positioned in a POPC bilayer, and MD was performed using both the open and the closed disulfide conditions. The tcl script described above was used to push water out of the membrane and MD simulation was performed for 200 ns.

### Preparation of the HC-HC structure based on Cx43-GJ structure

The HC-HC structure was prepared as above, however the HC structures were taken from the experimentally determined HC (PDB code: 7z23) structures and aligned to the template Cx26 GJC (PDB code: 2ZW3) [109]. After renumbering and aligning, a short minimization was performed in Maestro on all atoms, using the “minimize all” command. Subsequently, the model was submitted to OPM, and the Cx43 HC-HC model was prepared in VMD. Finally, MD was performed with open/closed disulfide bond conditions for 200 ns.

### Preparation and molecular dynamics of the cryo-EM based Cx43-GJ structure

The Cx43 GJC structure (PDB code 7z22) was prepared in VMD as above. The structure was split into 12 individual subunits. Cys disulfides were set between 54C(1) and 198C(3), 61C(2) and 192C(2) along with 65C(3) and 187C(1) using the DISU (disulfide) patch of VMD. The open S–S version was prepared without creating Cys disulfide bonds. The structure was positioned in two POPC bilayers and MD was performed using both the open and the closed disulfide conditions for 100 ns.

### Determination of stabilization centers

Stabilization centers (SCs) are pairs of residues which are primarily responsible for the stabilization of the structure by certain long range interactions [111]. Briefly, two residues are considered to form SC if they meet the following criteria: 1) they are separated by at least ten residues in the sequence; 2) at least one of their heavy-atom contact distances is less than the sum of the van der Waals radii of the two atoms, plus 1.0 Å; 3)both residues in the SC pair make at least seven contacts out of the possible nine contacts with the other residue and its four neighbours in the sequence in both directions. In order to identify SCs, MD trajectories from NAMD or Desmond were imported into VMD and individual frames at 10 ps interval were exported as pdb files. After adding sequence residues (SEQRES) data to the pdf files, SCs were identified using the SRide server [112]. Extracellular SCs were determined by selecting SCs containing at least one extracellular amino acid from EL1 residues 47–73 or EL2 residues 177–203 and appearing in at least 2% of the total running time. Atomic distances between SC forming residues during 100 ns MD of Cx43 HC-HC model are listed in SI Table S2. Minimum, maximum and average values are listed for all atom pairs that contribute to the SCs.

### Calculation of RMSD values

In order to calculate RMSD values, protein models in all frames of an MD simulation were aligned to the first frame (t = 10 ps) using the membrane segment of the protein (residues 21–46, 74–93, 156–176 and 204–229) as a base for alignment. After the alignment, RMSD changes of the extracellular part (residues 47–73 and 177–203) were calculated for all frames using VMD.

### Determination of H-bonds

Trans-GJ H-bonds were identified between residues 55–58 using the following donor atoms: backbone “N”, Asn “OD1”, Thr “OG1”, Gln “NE2” and the following acceptor atoms: backbone “O”, Asn “OD1”, Thr “OG1”, Gln “OE1”. Trans-GJ H-bonds were identified when the distance between donor hydrogen atoms and acceptor heavy atoms were below 2.5 Å. Cys-Cys H-bonds were identified between the Cys “SG” and Cys “HG1” atoms in the open S–S configurations. Threshold for Cys-Cys H-bond interactions was distance of 4.3 Å between sulphur and hydrogen atoms. H-bonds between Cys residues and residues 55–58 were identified between the donor and acceptor heavy atoms of residues 55–58 listed above and Cys “SG” atoms as either donor or acceptor. The criteria for identifying H-bonds were distance between sulphur and heavy atoms below 4.1 Å and distance between donor hydrogen and sulphur or other heavy atoms below 3.2 Å [86].

## Reporting summary

Gap junction (GJ) channels (GJCs) formed by members of connexin family proteins establish the cell-to-cell transfer of solutes via intercellular hemichannel (HC) docking. Here we validated mechanistic clues by in silico simulation of intercellular docking of homomeric Cx43 HCs. The HC docking process involves the emergence of trans-GJ interactions such as trans-GJ SC patterning and trans-GJ H-bond formation, triggered by the redox exchange of extracellular Cys disulfides. Significance of findings is that numerous GJCs in vertebrates may undertake intercellular HC docking similarly and may bring forward to the clinically-relevant GJC subtype-specific inhibition of HC docking.

### Supplementary Information

**Additional file 1: SI Table S1.** Structures of connexin proteins in the PDB database. **SI Table S2.** Intra-subunit SC distances between EL1 and EL2 in the Cx43HC-HC model. **SI Figure S1.** (A) Sequence alignment between Cx43 and Cx31.3 (PDB code: 6l3t) ^1^ generated by Swiss-Model. Transmembrane (TM) amino acids (AA) of Cx43 and Cx31.3 TM regions according to PPM are shown in red. TM AAs of Cx43 are as follows: 20-46, 74-94, 154-176, 205-226. (B) Sequence alignment between Cx43 and Cx26 (PDB code: 2zw3) 2 generated by Swiss-Model. TM amino acids (AA) of Cx43 and Cx26 TM regions according to PPM are shown in red. TM AAs of Cx43 are as follows: 20-46, 74-94, 154-176, 204-230. **SI Figure S2.** Comparison of Cx43 models to experimental Cx43 structures. **SI Figure S3.** Appearance of trans-GJ SCs in the Cx43HC-HC model after moving HCs away to 5 Å distance. **SI Figure S4.** Stabilization centers (SCs) in the Cx43HC-HC model are located near to extracellular cystines. **SI Figure S5.** Inter-subunit stabilization centers (SCs) within HCs are not disrupted by opening of disulfide bonds in the Cx43 HC-HC model.

## Data Availability

Cx26 GJC and Ca-free Cx31.3 HC structures used in our Cx43 homology models, as well as cryo-EM determined Cx43 HC and Cx43 GJC structures are freely available in the worldwide PDB (codes 2ZW3, 6L3T, 7Z23 and 7Z22 respectively; *see* SI Table S1). Raw MD data authenticating the findings of the manuscript can be downloaded at http://downloadables.ttk.hu/heja/ConnexinMD2023.
